# Spectral Feature Selection Optimization for Water Quality Estimation

**DOI:** 10.3390/ijerph17010272

**Published:** 2019-12-30

**Authors:** Manh Van Nguyen, Chao-Hung Lin, Hone-Jay Chu, Lalu Muhamad Jaelani, Muhammad Aldila Syariz

**Affiliations:** 1 Department of Geomatics, National Cheng Kung University, Tainan City 701, Taiwan; manh.ig239@gmail.com (M.V.N.); linhung@mail.ncku.edu.tw (C.-H.L.); aldilasyariz@gmail.com (M.A.S.); 2 Institute of Geography, Vietnam Academy of Science and Technology, 18 Hoang Quoc Viet, Vietnam; 3 Department of Geomatics Engineering, Institut Teknologi Sepuluh Nopember, Jawa Timur 60111, Indonesia; lmjaelani@geodesy.its.ac.id

**Keywords:** water quality mapping, Chl-a estimation model, multispectral satellite images, chlorophyll-a, inland turbid water

## Abstract

The spatial heterogeneity and nonlinearity exhibited by bio-optical relationships in turbid inland waters complicate the retrieval of chlorophyll-a (Chl-a) concentration from multispectral satellite images. Most studies achieved satisfactory Chl-a estimation and focused solely on the spectral regions from near-infrared (NIR) to red spectral bands. However, the optical complexity of turbid waters may vary with locations and seasons, which renders the selection of spectral bands challenging. Accordingly, this study proposes an optimization process utilizing available spectral models to achieve optimal Chl-a retrieval. The method begins with the generation of a set of feature candidates, followed by candidate selection and optimization. Each candidate links to a Chl-a estimation model, including two-band, three-band, and normalized different chlorophyll index models. Moreover, a set of selected candidates using available spectral bands implies an optimal composition of estimation models, which results in an optimal Chl-a estimation. Remote sensing images and in situ Chl-a measurements in Lake Kasumigaura, Japan, are analyzed quantitatively and qualitatively to evaluate the proposed method. Results indicate that the model outperforms related Chl-a estimation models. The root-mean-squared errors of the Chl-a concentration obtained by the resulting model (OptiM-3) improve from 11.95 $\mathrm{mg}\cdot m^{-3}$ to 6.37 $\mathrm{mg}\cdot m^{-3}$, and the Pearson’s correlation coefficients between the predicted and in situ Chl-a improve from 0.56 to 0.89.

## 1. Introduction

Detecting drastic changes in water quality is necessary to prevent unexpected environmental incidents. Conventional water sampling methods are reliable but are ineffective in identifying detailed spatial variations of water quality, which renders comprehensive management infeasible [1,2,3]. Remote sensing techniques have been proven effective in the selection of aquaculture sites and the qualitative measurement of regional water parameters, including suspended sediment, chlorophyll-a (Chl-a), and pollutant loads [4,5,6]. Kuhn et al. [7] used Landsat-8 and Sentinel-2 aquatic remote sensing reflectance products to estimate turbidity over the Amazon, Columbia, and Mississippi rivers. The ease of remote sensing techniques relies on the determination of the optical properties of water bodies. Phytoplankton and related materials, such as debris, heterotrophic organisms, and excreted organic matters, dominate the optical properties of waters in deep ocean waters; they are referred to as Case I waters whose optical properties vary with phytoplankton concentration [8]. The ratio of blue and green spectral reflectance has been proven a reliable measure for Chl-a concentration in Case I waters [9]. However, in most inland and coastal waters with high turbidity, which are referred to as Case II waters [8], optical properties are highly influenced by mineral particles, colored dissolved organic matters (CDOM), or microbubbles, apart from phytoplankton. The effect of the optical properties causes difficulty in differentiating phytoplankton from turbid waters [10]. The bio-optical relationship of Case II waters exhibits spatial nonlinearity and heterogeneity, which creates inaccuracy in the ratio of blue and green spectral reflectance for Chl-a concentration estimation [11,12,13].

Chl-a is an effective measure for estimating the nutritional status of a lake. From chlorophyll concentration, the status of eutrophication can be quickly assessed [14]. Numerous methods on the Chl-a concentration estimation of turbid inland waters have been proposed. These methods can be classified as empirical- and analytical-based methods. Analytical-based methods analyze the physical interconnections among absorption, scattering coefficients, and water parameters at different wavelengths of spectral bands, based on the radiative transfer equation [3,15,16,17]. By contrast, empirical-based methods address the link between spectral bands of satellite images and measured water parameters of interest [12,13,18,19,20]. Recently, a neural network was also applied to define the various eutrophic levels and estimate the water quality parameters [21,22]. Statistical techniques are leveraged on empirical-based methods to relate water quality observations directly to remotely sensed spectral information [23]. The three- or two-band reflectance model was originally developed to estimate the Chl-a concentration of terrestrial vegetation [12,24]. The three- or two-band reflectance model has been widely used to estimate Chl-a in turbid waters using the reflectances in the near-infrared (NIR) band (710 and 750 nm) and red band (near 670 nm) [20]. In addition, Mishra and Mishra [25] proposed a normalized difference chlorophyll index (NDCI), which is based on the normalized differences between two spectral bands, to estimate Chl-a concentration; Han and Rundquist [26] and Moses et al. [18] introduced another two-band model using near-infrared (NIR) and red spectral bands. The two-band, three-band, and NDCI models have demonstrated good performances in Chl-a concentration estimation. However, the selection of appropriate spectral bands in the model for the mapping and estimation of water quality in various water environments remain challenging [1]. These methods are simple and efficient, but they utilize solely NIR–red spectral regions and do not search for the optimal model [27,28].

This study proposes an optimization process for spectral feature selection in water quality estimation. The proposed model is a combination of empirical models with optimal spectral bands. A set of feature candidates is generated by following the knowledge of two-band, three-band, and NDCI models with available spectral bands. Moreover, the spatial pattern of water quality can be estimated on the basis of the optimal features. The remainder of this paper is organized as follows: Section 2 introduces the study area and datasets; Section 3 presents the methodology; Section 4 displays the experimental results; Section 5 shows the detailed discussion; and Section 6 provides the conclusion and future works.

## 2. Materials and Study Area

**Figure 1:**
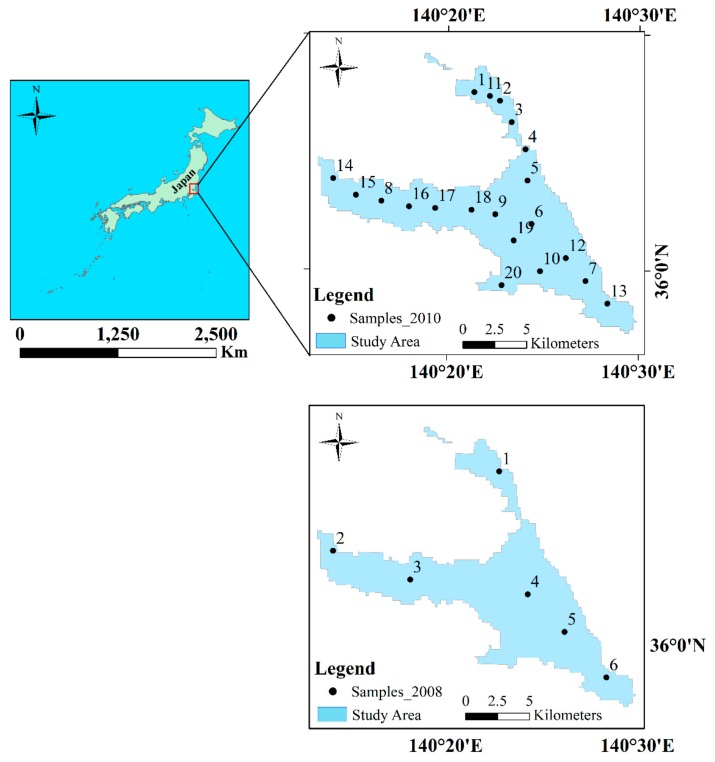
Study area and locations of in situ samples in 2008 and 2010.

**Figure 2:**
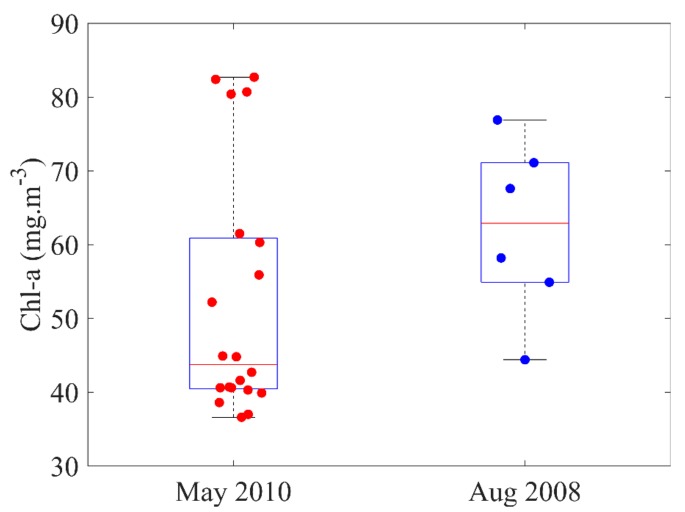
Box-plots of the summary statistics for chlorophyll-a (Chl-a) in 2008 and 2010.

Lake Kasumigaura (36°09′ N, 140°14′ E) is the second largest lake in Japan a 220 km^2^ surface area and 4.0 m average depth. The in situ samples collected by the University of Tsukuba in 2008 and 2010 were utilized, respectively (Figure 1). The acquisition dates of the in situ samples coincided with those of the medium-resolution imaging spectrometer (MERIS) images. Water sample collections were performed between 10:00 and 14:00 h local time over optically deep waters. They were kept in ice boxes and taken to the laboratory. Chlorophyll-a was extracted using methanol (100%) at 4 °C under dark conditions for 24 h. The optical density of the extracted chlorophyll-a was measured at four wavelengths (750, 663, 645, and 630 nm), and the concentration was calculated according to SCOR-UNESCO equations [29]. Following the sample filtering strategy in [30], several in situ samples were regarded as outliers using the standard deviation of the difference between the actual Chl-a concentration and predicted Chl-a concentration. A total of 26 in situ samples remain after outlier filtering. The descriptive statistics shows extensive variation in the Chl-a concentration ranges, 4.40 (min), 76.90 (max), 62.90 (median) $\mathrm{mg}\cdot m^{-3}$ and 36.60 (min), 83.40 (max), 44.80 (median) $\mathrm{mg}\cdot m^{-3}$ in 2008 and 2010, respectively (Figure 2). The Chl-a concentration is high in upstream areas where Lake Kasumigaura receives high-turbidity waters from two narrow rivers, including Sakura River and Koise River; Lake Kasumigaura is under the influence of agricultural activities. During the monsoon season, which is generally in May, the fresh water inflow lowers the Chl-a concentrations [31].

The in situ samples were divided into two sets, namely, training and testing. The first set with 10 samples in 2010 was used for feature candidate optimization and training, and the second set with the remaining 10 samples in 2010 and 6 samples in 2008 was used for testing. In addition, The MERIS images were atmospherically corrected using the method in [32]. To ensure that the water pixels were neither mixed with land pixels nor contaminated by clouds, data collected less than one MERIS pixel from the bank and/or covered by clouds were excluded. Moreover, MERIS has 15 narrow spectral bands in the visible and NIR spectral ranges [33]. The reflectances of 14 narrow spectral bands were used for feature generation and selection without considering $B_{15}\left(900\right)$.

## 3. Methods

**Figure 3:**
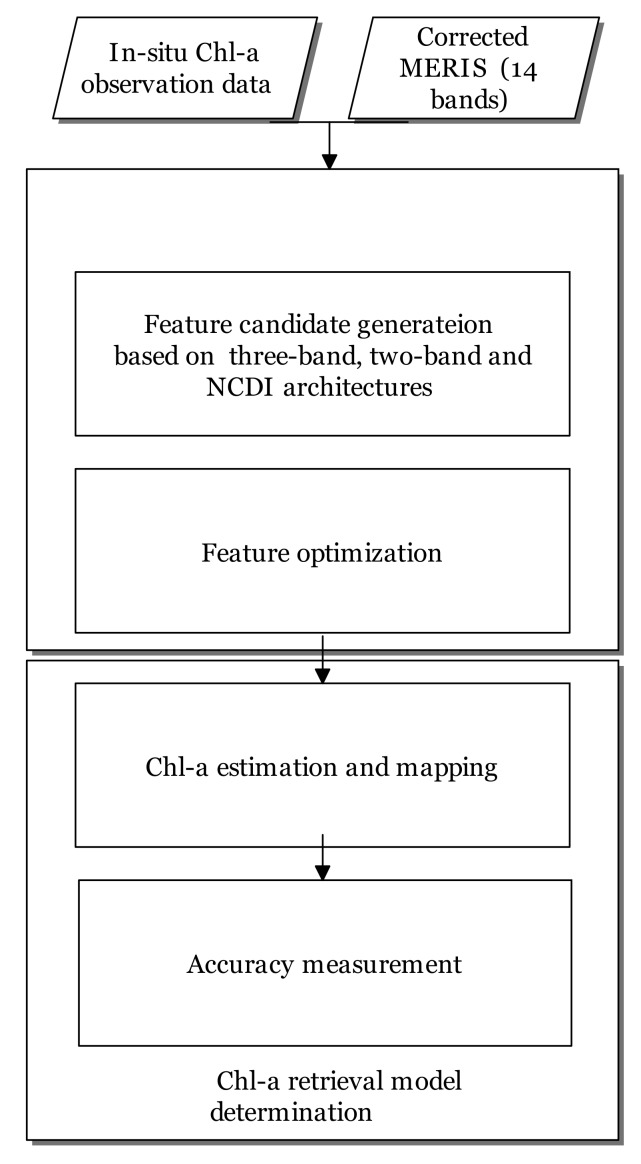
Procedures of the study, including feature candidate generation from three-band, two band, and the normalized difference chlorophyll index (NDCI), as well as feature optimization and Chl-a retrieval model determination.

The workflow of the proposed method consisted of three main steps, namely, feature candidate generation, candidate optimization, and Chl-a retrieval model determination (Figure 3). The inputs to the method were the remote sensing reflectance $R_{rs}(\lambda )$ of MERIS images and their corresponding in situ Chl-a measurements. In the first step, a set of feature candidates formed from the two-band, three-band, and NDCI models, was generated. Next, candidate optimization based on neighborhood component analysis [34] was performed in the second step to determine the significances of feature candidates. In the third step, a multivariate linear regression was conducted with the optimal determined features to determine the Chl-a estimation model. Section 3.1 and Section 3.2 introduce feature candidate generation and feature optimization, respectively. Section 3.3 presents Chl-a retrieval model determination, mapping, and validation.

### 3.1. Feature Candidate Generation

The candidates were generated from two-band, three-band, and NDCI models. These three models are briefly introduced. The three-band model based on NIR and red spectral bands was proposed by Dall’Olmo and Gitelson [35]. The model is based on the fact that the difference in reciprocal surface reflectance $R_{rs}{\left(\lambda_{1}\right)}^{-1}$ and $R_{rs}{\left(\lambda_{2}\right)}^{-1}$ on two spectral wavelengths $\lambda_{1}$ and $\lambda_{2}$ must be small to omit the absorption of suspended solids and CDOM. In addition, this model assumes that the total absorption of Chl-a, CDOM, and total suspended solids beyond the spectral wavelength of 730 nm is nearly zero, and the back-scattering coefficient of Chl-a is spectrally invariant. Given these facts and assumptions, the structure of three-band model is defined as

(1) $$\left[R_{\mathrm{rs}}^{-1}\left(\lambda_{1}\right)-R_{\mathrm{rs}}^{-1}\left(\lambda_{2}\right)\right]\times R_{rs}\left(\lambda_{3}\right).$$

Dall’Olmo et al. [12,35] suggested setting the wavelength $\lambda_{1}$ to the red spectral region between 660 and 690 nm to maximize the sensitivity to the changes in Chl-a concentrations, setting the wavelength $\lambda_{2}$ to the range between 690 and 730 nm; the aim is to remove the influence of other absorption factors, such as tripton and CDOM, and locating the wavelength $\lambda_{3}$ in the range between 730 and 760 nm to eliminate misestimation caused by particulate backscattering. The structure of the two-band model is defined as

(2) $$R_{\mathrm{rs}}^{-1}\left(\lambda_{1}\right)\times R_{\mathrm{rs}}\left(\lambda_{2}\right).$$

Moses et al. [18] presented another two-band algorithm to retrieve the Chl-a of Case II waters. The model is formulated as $R_{rs}^{-1}\left(665\right)\times R_{rs}\left(709\right)$ to match the designed bands of the MERIS sensor. The λ_3_ is set to 709 nm instead of 753 nm because of the following: (1) The wavelength at 709 nm can better represent the chlorophyll-induced reflectance than that at 753 nm. (2) The magnitude of the water-leaving radiance at 753 nm is lower than that at 709 nm given the increased absorption by water at long wavelengths. Thus, the uncertainties of the atmospheric correction procedure attributed to a low signal-to-noise ratio are less amplified at 709 nm than at 753 nm. (3) $\lambda_{3}$ = 708 nm is close to $\lambda_{1}$ = 665 nm. Thus, the atmospheric effect at 709 nm is closer to that at 665 nm. This characteristic reduces the sensitivity of the two-band model with $\lambda_{3}$ at 709 nm to spectral non-uniform atmospheric effects. Mishra and Mishra [25] developed NDCI to estimate Chl-a concentration in turbid waters. This method utilizes the spectral main absorption peak in the red spectral region at 665 and 709 nm. The NDCI is formulated as the normalized spectral difference between $R_{rs}\left(709\right)$ and $R_{rs}\left(665\right)$; that is,$\left[R_{rs}\left(665\right)-R_{rs}\left(709\right)\right]/\left[R_{rs}\left(665\right)+R_{rs}\left(709\right)\right]$. Thus, the measurement form is represented by

(3) $$\left[R_{rs}\left(\lambda_{1}\right)-R_{rs}\left(\lambda_{2}\right)\right]/\left[R_{rs}\left(\lambda_{1}\right)+R_{rs}\left(\lambda_{2}\right)\right].$$

The current study selected the four bands $B_{7}\left(665\right),\cdots ,\;B_{10}\left(754\right)$ as the common bands as suggested in previous studies [13,18,25,36]. The following were the priority choices: four common spectral regions, namely, the 7th–10th spectral bands of MERIS images with wavelength centers of 665, 681, 709, and 754 nm (denoted as $B_{7}\left(665\right)$, $B_{8}\left(681\right)$, $B_{9}\left(709\right)$, and $B_{10}\left(754\right)$, respectively), and one band from the remaining bands. In each candidate set, five bands were selected and the feature candidates based on the three models were generated. Table 1 shows the examples of feature candidate generation. A total of 30 possible feature candidates were generated by using the three-band model in Equation (1). A total of 10 possible feature candidates were generated by using the two-band model in Equation (2). A total of 10 possible feature candidates were generated by using the NDCI in Equation (3). In total, 50 possible candidates were generated.

### 3.2. Feature Optimization

Feature optimization is performed to select substantial candidates from the candidate pool $\left\{C_{1},\cdots ,C_{n_{c}}\right\}$ (where $n_{c}$ represents the number of candidates) such that the selected candidates are sensitive to the changes in Chl-a concentration and are effective in Chl-a concentration estimation. The candidate sample vector $x_{i}:\left\{x_{i,1},\cdots ,x_{i,n_{c}}\right\}$ is defined. $x_{i,j}$ belongs to the candidate model $C_{j}$ for the *i*-th in situ sample (denoted as $S_{i}$). The in situ sample $S_{i}$ is represented as a pair $\left(x_{i},y_{i}\right)$, where $y_{i}\in R$ denotes the Chl-a value of sample $S_{i}$. Candidate selection is based on neighborhood component analysis [34], which is a nonparametric classification and feature selection method. The optimization aims to identify substantial values for each candidate. Given a set of training data $T\;=\;\left\{S_{1}:\left(x_{1},y_{1}\right),\cdots ,S_{n_{s}}:\left(x_{n_{s}},y_{n_{s}}\right)\right\}$ containing $n_{s}$ samples, the optimization aims to find a substantial value for each candidate x. The procedure begins with the selection of a sample from *T* as the reference sample, which is denoted as $S_{r}:\left(x_{r},y_{r}\right)$, and the weighted distance is calculated between the reference sample and other samples using

(4) $$w\text{\_}dist\left(x_{i},x_{r}\right)\;=\;\overset{n_{c}}{\underset{j\;=\;1}{\sum }}w_{j}\left|x_{i,j}-x_{r,j}\right|,$$

where $w\text{\_}dist\left(x_{i},x_{r}\right)$ represents the weighted distance between $x_{i}$ and $x_{r}$, and $w_{j}$ denotes the weight and significance of the feature candidate $C_{j}$ that the optimization wishes to obtain. A leave-one-out strategy is adopted to predict the response for reference $x_{r}$ by using the dataset $T-\left\{S_{r}:\left(x_{r},y_{r}\right)\right\}$; that is, the training set T excluding the reference sample $\left(x_{r},y_{r}\right)$, to obtain the weights and to define the objective function of the optimization. Next, the probability of using $x_{i}$ in the prediction of reference $x_{r}$ is defined as measuring a normalized distance between these two samples with a Gaussian kernel function; that is,

(5) $$p_{ir}\left(x_{1},\cdots ,x_{n_{s}}\right)\;=\;g\left[w\text{\_}dist\left(x_{i},{\;x}_{r}\right)\right]/\overset{n_{s}}{\underset{j\;=\;1}{\sum }}g\left[w\text{\_}dist\left(x_{i},x_{j}\right)\right],$$

where $g\left(\cdot \right)$ represents the Gaussian kernel function. Given these probabilities, the cost function $f_{r}\left(S_{1},\cdots ,S_{n_{s}}\right)$ for the reference sample is defined as the summation of the loss caused by the response of the reference sample and that of other samples multiplied by their probability; that is,

(6) $$f_{r}\left(S_{1},\cdots ,S_{n_{s}}\right)\;=\;\sum_{i\;=\;1,\;i\neq r}^{n_{s}}p_{ir}l(y_{i},y_{r}),$$

where $l\left(y_{i},y_{r}\right)$ represents a loss function that measures the similarity between the response $y_{i}$ in the sample $S_{i}$ and the response $y_{r}$ in the reference sample $S_{r}$. The loss function is formulated as $l\left(y_{i},y_{r}\right)\;=\;\left|y_{i}-y_{r}\right|$ in the implementation.

The overall objective function is obtained by summing the cost function from each reference sample. In addition, a regularization term is introduced to the optimization to avoid overfitting. The objective function can be formulated combining these two terms. Considering the objective function, the optimal weights are defined as

(7) $$\tilde{w}=\arg\underset{w\;=\;\left\{w_{1},\cdots ,w_{n_{c}}\right\}}{\min}\left\{\sum_{r\;=\;1}^{n_{s}}f_{r}\left(S_{1},\cdots ,S_{n_{s}}\right)+\alpha \times \left(w_{1}^{2}+\cdots +w_{n_{c}}^{2}\right)\right\},$$

where α is the parameter for balancing the fitness of the cost functions and the smoothness of the obtained weights. The optimization in Equation (7) is solved to search for the optimal weights $\tilde{w}$ by using the gradient descent method [37], which is a commonly used optimization solver that iteratively moves toward the optimal solution from an initial solution in search space with the aid of the gradient direction of the objective function.

### 3.3. Chl-A Estimation, Mapping, and Validation

After determining the weights in the candidates, the optimal feature can be found. The relation between Chl-a concentrations and the optimal features is identified using the regression model, and the spatial pattern of Chl-a concentration is estimated on the basis of the best regression model with optimal features.

To evaluate the generated and related Chl-a estimation models, the commonly used measurements, including the slope of the regressed line denoted by m, the root-mean-square error (RMSE), and Pearson’s correlation coefficient denoted by r between the estimated and measured Chl-a, were adopted as

(8) $$\mathrm{RMSE}\;=\;\sqrt{\frac{\sum_{i\;=\;1}^{n_{v}}{\left(chla_{i}^{p}-chla_{i}^{m}\right)}^{2}}{n_{v}}},$$

(9) $$r=\frac{\sum_{i\;=\;1}^{n_{v}}\left(chla_{i}^{p}-\;\bar{chla^{p}}\right)\left(chla_{i}^{m}-\;\bar{chla^{m}}\right)}{\sqrt{\left(\sum_{i\;=\;1}^{n_{v}}{\left(chla_{i}^{p}-\;\bar{chla^{p}}\right)}^{2}\right)\left(\sum_{i\;=\;1}^{n_{v}}{\left(chla_{i}^{m}-\;\bar{chla^{m}}\right)}^{2}\right)}},$$

(10) $$m=\frac{\sum_{i\;=\;1}^{n_{v}}\left(chla_{i}^{p}-\;\bar{chla^{p}}\right)\left(chla_{i}^{m}-\;\bar{chla^{m}}\right)}{\sum_{i\;=\;1}^{n_{v}}{\left(chla_{i}^{p}-\;\bar{chla^{p}}\right)}^{2}},$$

where $chla_{i}^{p}$ and $chla_{i}^{m}$ represent the predicted and measured Chl-*a* concentration of sample $S_{i}$, respectively;$\bar{chla^{p}}$ and $\bar{chla^{m}}$ denote the average predicted and average measured Chl-*a* concentration, respectively; and $n_{v}$ represents the number of testing samples. The RMSE indicates the absolute fit of the model to the data; that is, how close the observed data points are to the model’s predicted values. The correlation and the slope of the regression line were defined as the statistical association between observation and prediction. The better model exists in the lower RMSE, with a higher correlation between observation and prediction and the 1:1 slope of the regression line between observation and prediction.

## 4. Results

### 4.1. Results of Feature Optimization

The Chl-a estimation models were generated by the proposed method from the candidate sets (OptiM-1–OptiM-5) and the related methods from the two-band model [18] (denoted as TwoB-M), three-band model [13] (denoted as ThreeB-G), and NDCI model [25] in Table 2. Table 3 shows the regression models between the Chl-a concentrations and spectral features. The Chl-a in situ samples from Lake Kasumigaura and MERIS images with the same acquisition data were used as test data, and the coefficient of determination $R^{2}$ was adopted as the measurement of regression fitness. The $R^{2}$ of estimation results from the resulting models are between 0.57 and 0.62, which are superior to those from the two-band model, three-band model, and NDCI model [25] ($R^{2}\;=\;0.44-0.55$). Based on these measurements, the optimal model is OptiM-3, which contains two candidates in the form of a three-band model; that is, $\left[R_{\mathrm{rs}}^{-1}\left(665\right)-R_{\mathrm{rs}}^{-1}\left(709\right)\right]\times R_{\mathrm{rs}}\left(510\right)$ and $\left[R_{\mathrm{rs}}^{-1}\left(665\right)-R_{\mathrm{rs}}^{-1}\left(510\right)\right]\times R_{\mathrm{rs}}\left(709\right)$.

**Figure 4:**
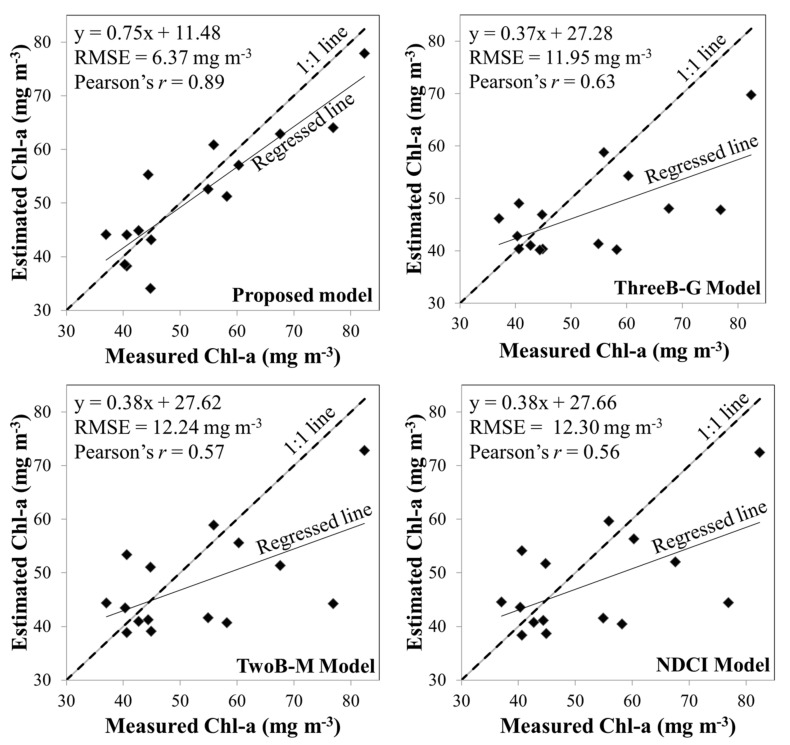
Comparison between estimated and measured chlorophyll-a (Chl-a) concentration provided by compared models in OptiM-3, ThreeB-G, TwoB-M, and NDCI models.

Table 4 shows the model performance comparisons for validation. This result agrees with the conclusions of previous studies [38,39] and indicates that the performance of three-band model is slightly better than that of two-band models and NDCI. In addition, the comparisons show that the RMSE of the best model is 6.37 $\mathrm{mg}\cdot m^{-3}$ (Figure 4). By contrast, the RMSEs of the related previous models (ThreeB-G, TwoB-M, NDCI) are close to 12 $\mathrm{mg}\cdot m^{-3}$ (Figure 4). The combination of these two three-band candidates outperforms the three-band model with optimal bands [13]. Therefore, the obtained model can preserve the characteristics of the three-band model while optimally estimating Chl-a concentrations.

### 4.2. Mapping with Various Spectral Features

**Figure 5:**
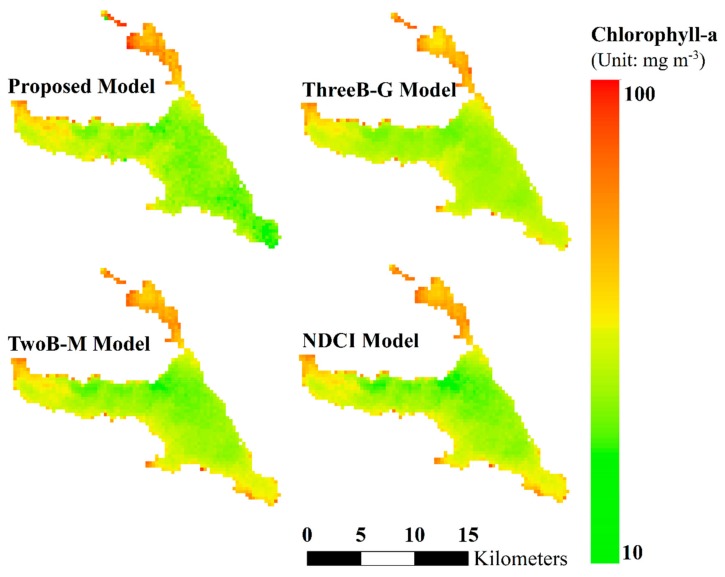
Maps of spatial distribution of Chl-a in 2010 generated by our proposed model (OptiM-3), ThreeB-G [13], TwoB-M [18], and NDCI [25] (unit: $\mathrm{mg}\cdot m^{-3}$).

Chl-a concentration maps are generated by the resulting model and the related empirical models in Figure 5. Spatial patterns of Chl-a in the four maps are similar. The Chl-a concentration is relatively low in the southern area in the map generated by our model compared with that generated by the compared models, especially the regions near the lake boundaries. In addition, the map generated by our model is spatially smoother than the compared model, and the spatial distribution of Chl-a concentration in our map is more fitted with the result in [40]. Moreover, the Chl-a concentration map can be used to identify the Chl-a hotspot in the lake. For instance, a high Chl-a concentration can be found at the northern part of the lake. The selection of appropriate features is complex and challenging because the changes in the chemical and physical properties of water can lead to different model/feature determination. This study provides an accurate satellite Chl-a model of turbid water by using optimal feature generation and selection based on feature generation from the two-band, three-band, and NDCI models. The regional and spatial information of Chl-a concentration can be generated considering a model with such satellite information.

## 5. Discussion

The model for optimal feature selection is based on feature generation from the two-band, three-band, and NDCI models. This study can eventually provide an accurate satellite Chl-a model of turbid productive (Case II) water by conducting empirical and optimal feature generation and selection.

The optical properties in clear waters are controlled by phytoplankton. Chl-a retrieval in clear waters is commonly used at the blue and green spectral regions, whereas Chl-a retrieval in turbid waters shifts from the blue and green to the red and NIR spectral regions to avoid high absorption of CDOM and non-algal particles [41]. However, changes in Chl-a concentration are sensitive at the red region between 660 and 690 nm [13]. The wavelength at 708 nm fully represents the Chl-a-induced reflectance peak in the NIR, whereas the reflectance at 753 nm does not because it mostly depends only on the scattering of suspended particles. The commonly used models [42] consider the following ratios: first, reflectances at the blue region (440–510 nm) within the first peak of strong absorption to reflectances at the green region (550–555 nm) with the minimum absorption [43]; and second, reflectances at the NIR region (685–710 nm) with the minimum absorption to reflectances at the red region (670–675 nm) with the second peak of absorption [44]. In this paper, the features from the models typically include blue, red, and NIR spectral regions and are highly related to reflectances within the first peak of strong absorption at the blue region to reflectances at the second peak absorption and minimum absorption at the red and NIR regions (665 and 709 nm). The features from the existing models correlate with the reflectances at the red and NIR regions at 620, 681, and 709 nm. However, the existing models [13,18,25,45] are between the red and NIR spectral regions. This result matches previous results [13,27], showing that the NIR spectral regions are negligibly affected by the presence of particles and CDOM in the estimation of Chl-a concentrations [28]. The model obtains the three-band features based on our schemes, and its accuracy is higher than those of the existing widely applied empirical algorithms from previous studies. The selection of appropriate features is complex and challenging due to the changes in chemical and physical properties of water.

This study primarily applies feature selection optimization to satellite-based water quality mapping. Selecting the important features in the feature selection algorithm aims to derive accurate predictive models for the estimation of Chl-a concentration. The optimal feature selection is useful for determining site-specific and generally used parameters for Chl-a estimation. From the selected features, the band at 709 nm is commonly selected in the models. The radiance peak at 709 nm in water-leaving radiance, that is, the MERIS maximum chlorophyll index, is extensively used to measure the presence of high Chl-a concentration against a scattering background [13]. Moreover, the Chl-a concentration map can be used to identify the Chl-a hotspot in the lake. The high Chl-a in the water environments becomes warmer in the summer, leading to increased algal growth rates. For example, high Chl-a concentration can be found at the northern part of the lake. The regional and spatial information of Chl-a concentration cannot be generated without such satellite information and modeling. In addition, the selected model will affect the spatial pattern of Chl-a estimation. The Chl-a concentration in the proposed model is lower in the southern area than those in the previous models, especially in the boundary of the lake.

## 6. Conclusions

This study provides a systematic approach for water quality estimation based on optimal feature generation and selection and proposes an optimization of feature generation and selection for the determination of a Chl-a concentration model. A set of candidates was generated on the basis of the two-band, three-band, and NDCI models. The optimal model, which consists of one or several candidates with substantial weights, was determined through neighborhood component analysis with an objective function. In situ samples from Lake Kasumigaura, Japan, and MERIS images were used to test the feasibility of the proposed process. The Chl-a concentration estimation performance of the obtained model was compared with that of related models. 

The model can successfully estimate Chl-a concentrations from optimal spectral features. However, the geographical and seasonal variations in the environments of turbid inland waters complicate the selection of spectral bands used in the empirical models. The combination of spectral bands is identified as the optimal features using the proposed optimization. Quantitative measurements, including RMSE, r, and *m*, demonstrate the superiority of the obtained optimal model over the previous related models. In future work, images from Sentinel 3, a successor of MERIS, and additional in situ Chl-a samples will be utilized. Moreover, a nonlinear estimation model will be developed by using an artificial neural network.

## Figures and Tables

**Table 1:** Example of feature candidate generation from the selected bands.

Selected Spectral Bands	Possible Feature Candidates	Models	Of Candidates	Notation
$B_{6}\left(620\right)$ $B_{7}\left(665\right)$ $B_{8}\left(681\right)$ $B_{9}\left(709\right)$ $B_{10}\left(754\right)$	$\left[R_{\mathrm{rs}}^{-1}\left(620\right)-R_{\mathrm{rs}}^{-1}\left(665\right)\right]\times R_{rs}\left(681\right)$ $\left[R_{\mathrm{rs}}^{-1}\left(681\right)-R_{\mathrm{rs}}^{-1}\left(709\right)\right]\times R_{rs}\left(754\right)$	Three-band model (Equation (1))	30	$\begin{array}{c} C_{1}\\ \vdots \\ C_{30} \end{array}$
	$R_{\mathrm{rs}}^{-1}\left(620\right)\times R_{\mathrm{rs}}\left(665\right)$ $R_{\mathrm{rs}}^{-1}\left(620\right)\times R_{\mathrm{rs}}\left(681\right)$	Two-band model (Equation (2))	10	$\begin{array}{c} C_{31}\\ \vdots \\ C_{40} \end{array}$
	$\left[R_{\mathrm{rs}}\left(620\right)-R_{\mathrm{rs}}\left(665\right)\right]\times {\left[R_{\mathrm{rs}}\left(620\right)+R_{\mathrm{rs}}\left(665\right)\right]}^{-1}$, $\left[R_{\mathrm{rs}}\left(665\right)-R_{\mathrm{rs}}\left(681\right)\right]\times {\left[R_{\mathrm{rs}}\left(665\right)+R_{\mathrm{rs}}\left(681\right)\right]}^{-1},$	NDCI (Equation (3))	10	$\begin{array}{c} C_{41}\\ \vdots \\ C_{50} \end{array}$

**Table 2:** Proposed and related Chl-a estimation models.

Model Name	Model Feature
OptiM-1	$\left\{\left[R_{\mathrm{rs}}^{-1}\left(665\right)-R_{\mathrm{rs}}^{-1}\left(709\right)\right]\times R_{\mathrm{rs}}\left(681\right)\right\}.$
OptiM-2	$\left\{\left[R_{\mathrm{rs}}^{-1}\left(665\right)-R_{\mathrm{rs}}^{-1}\left(709\right)\right]\times R_{\mathrm{rs}}\left(490\right)\right\}$
OptiM-3	$\left\{\begin{array}{c} \left[R_{\mathrm{rs}}^{-1}\left(665\right)-R_{\mathrm{rs}}^{-1}\left(709\right)\right]\times R_{\mathrm{rs}}\left(510\right),\\ \left[R_{\mathrm{rs}}^{-1}\left(665\right)-R_{\mathrm{rs}}^{-1}\left(510\right)\right]\times R_{\mathrm{rs}}\left(709\right) \end{array}\right\}$
OptiM-4	$\left\{\begin{array}{c} \left[R_{\mathrm{rs}}^{-1}\left(665\right)-R_{\mathrm{rs}}^{-1}\left(560\right)\right]\times R_{\mathrm{rs}}\left(681\right),\\ \left[R_{\mathrm{rs}}^{-1}\left(709\right)-R_{\mathrm{rs}}^{-1}\left(560\right)\right]\times R_{\mathrm{rs}}\left(681\right) \end{array}\right\}$
OptiM-5	$\left\{\left[R_{\mathrm{rs}}^{-1}\left(709\right)-R_{\mathrm{rs}}^{-1}\left(620\right)\right]\times R_{\mathrm{rs}}\left(681\right)\right\}$
ThreeB-G [13]	$\{[R_{\mathrm{rs}}^{-1}\left(665\right)-R_{\mathrm{rs}}^{-1}\left(709\right)]\times R_{\mathrm{rs}}\left(754\right)$}
TwoB-M [18]	$\{\left[R_{\mathrm{rs}}\left(709\right)\times R_{\mathrm{rs}}^{-1}\left(665\right)\right]$}
NDCI [25]	$\left\{\left[R_{\mathrm{rs}}^{-1}\left(709\right)-R_{\mathrm{rs}}^{-1}\left(665\right)\right]/\left[R_{\mathrm{rs}}^{-1}\left(709\right)-R_{\mathrm{rs}}^{-1}\left(665\right)\right]\right\}$

**Table 3:** Chl-a estimation models using regression fitness. The intercept and two slopes of the regression lines are denoted as $a_{0}$, $a_{1}$, and $a_{2}$, respectively.

Models	$a_{0}$	$a_{1}$	$a_{2}$	$R^{2}$
OptiM-1	0.77	235.32	–	0.57
OptiM-2	1.93	174.26	–	0.61
OptiM-3	−7.74	94.03	106.40	0.61
OptiM-4	−174.57	691.61	−280.42	0.62
OptiM-5	48.51	−183.61	–	0.59
ThreeB-G [13]	24.91	115.14	–	0.44
TwoB-M [18]	−87.93	103.95	–	0.55
NDCI [25]	9.17	295.02	–	0.55

**Table 4:** Comparison of performance of Chl-a estimation models using the RMSE, Pearson’s correlation coefficient, and slope *m.*

Models	RMSE $\left(mg\cdot m^{-3}\right)$	Pearson’s Coefficient	*m*
No. of testing samples (*n* = 16)			
OptiM-1	11.91	0.58	0.40
OptiM-2	9.14	0.77	0.57
OptiM-3	6.37	0.89	0.75
OptiM-4	13.65	0.52	0.56
OptiM-5	9.56	0.71	0.54
ThreeB-G [13]	11.95	0.63	0.37
TwoB-M [18]	12.24	0.57	0.38
NDCI [25]	12.30	0.56	0.38

OptiM-3: Optimal resulting model.
